# Supplementary material for: Withdrawing Antipsychotics for Challenging Behaviours in Adults with Intellectual Disabilities: Experiences and Views of Prescribers
Source: Int J Environ Res Public Health. 2022 Dec 19;19(24):17095. doi: 10.3390/ijerph192417095 (PMC9779134; doi:10.3390/ijerph192417095)
Supplement: Supplementary file 1 [file ijerph-19-17095-s001.zip › ijerph-1992619-supplementary/Supplementary File S1. Survey among prescribers of psychotic drugs to people with intellectual disabilities.docx]

Questionnaire about experiences with the deprescribing of antipsychotic drugs; a survey among prescribers of psychotic drugs to people with intellectual disabilities (ID) in the Netherlands (2021)

Of note: We left out the last two administrative questions.

**Q1.** What is your current position?

a) consultant; b) psychiatrist, intellectual disability (ID) physician, general practitioner or specialist nurse; c) trainee- psychiatrist, - ID physician, - general practitioner or - specialist nurse, d) other / if yes: specify

**Q2.** Where do you practice?

a) institutional care or cure / if yes: specify; b) own practice / if yes: specify

**Q3.** In which province are you working?

**Q4.** How many years is your working experience in ID healthcare?

a) less than 10 years, b) 10-19 years, c) 20-29 years, d) more than 30 years.

**Q5.** Do you know the number of individuals with an ID in your practice? Could you provide an estimate?

a) yes (please specify the number and percentage of the total number of your patients), b) not applicable, because I am an ID physician and all my patients are intellectually disabled, c) don’t know

**Q6.** Do you know or could you estimate the percentage of patients with ID in your practice who use antipsychotics?

a) 0-25%, b) 25-50%, c) 50-75%, d) 75-100%, e) don’t know

**Q7.** Do you know or could you estimate the percentage of patients with ID in your practice who use antipsychotics without a proper psychiatric indication?

a) 0-25%, b) 25-50%, c) 50-75%, d) 75-100%, e) don’t know

**Q8.** Do you prescribe antipsychotics to individuals with ID or do you prescribe repeat medication?

a) yes, b) no

**Q9.** When did you start to deprescribe antipsychotics for challenging behaviour to individuals with ID in your practice?

a) not yet, b) less than 1 year ago, c) 1-3 years ago, d) 3-5 years ago, e) more than 5 years ago

**Q10.** At what percentage of the antipsychotic drug users for challenging behaviours with ID in your practice did you try to deprescribe antipsychotics?

a) 0%, b) 1-25%, c) 26-50%, d) 50% and over, e) don’t know / no data available, f) other / if yes: specify

**Q11.** At what percentage of the antipsychotic drug users for challenging behaviours with ID in your practice did you successfully deprescribe all antipsychotics?

a) 0%, b) 1-25%, c) 26-50%, d) 50% and over, e) don’t know / no data available, f) other / if yes: specify

**Q12.** At what percentage of the antipsychotic drug users for challenging behaviours with ID in your practice did you achieve a dose reduction in antipsychotic drug use of over 50%?

a) 0%, b) 1-25%, c) 26-50%, d) 50% and over, e) don’t know / no data available, f) other / if yes: specify

**Q13.** How much time do you take for the process of deprescribing antipsychotics for challenging behaviour in patients with ID?

a) 0-6 months, b) 6-12 months, c) 6-18 months, d) more than 18 months

**Q14.** Complete withdrawal of antipsychotics for challenging behaviour is achieved in a part of patients. In a part of those who achieved complete withdrawal the antipsychotics drug use must be reinstated. At what percentage of your patients who achieved complete withdrawal was the antipsychotic drug use reinstated? What was the time between complete withdrawal and the reinstatement?

a) 0% within 3 months, a1) 1-25% reinstatement within 3 months, a2) 25-50% reinstatement within 3 months, a3) over 50% reinstatement within 3 months

b) 0% within 6 months, b1) 1-25% reinstatement within 6 months, b2) 25-50% reinstatement within 6 months, b3) over 50% reinstatement within 6 months

c) 0% within 12 months, c1) 1-25% reinstatement within 12 months, c2) 25-50% reinstatement within 12 months, c3) over 50% reinstatement within 12 months

**Q15.** At what percentage of those patients with ID in your practice who tried to withdraw their antipsychotics a serious and persistent worsening in psychiatric or behavioural condition occurred?

a) 0%, b) 1-25%, c) 26-50%, d) 50% and over, e) don’t know / no data available, f) other / if yes: specify

**Q16.** At what percentage of those who had such a serious event, this was a reason to slow down the withdrawal process, but ultimately achieved complete withdrawal?

a) 0%, b) 1-25%, c) 26-50%, d) 50% and over, e) don’t know / no data available

**Q17.** At what percentage of those who had such a serious event, this was a reason to slow down the withdrawal process, but achieved no complete withdrawal?

a) 0%, b) 1-25%, c) 26-50%, d) 50% and over, e) don’t know / no data available

**Q18.** Where there any complications during the withdrawal of antipsychotics in your patients with ID? Multiple answers are possible.

a) increase in challenging behaviours, b) withdrawal symptoms, c) unmasking of previously hidden psychiatric symptoms, d) other/ if yes: specify.

**Q19.** At what percentage were these complications reason to slow down the withdrawal process after which complete withdrawal was achieved?

a) 0%, b) 1-25%, c) 26-50%, d) 50% and over, e) don’t know / no data available

**Q20.** At what percentage were these complications reason to slow down the withdrawal process after which complete withdrawal was NOT achieved?

a) 0%, b) 1-25%, c) 26-50%, d) 50% and over, e) don’t know / no data available

**Q21.** Were there certain patient groups in which withdrawal was easy and with no or few withdrawal symptoms? Multiple answers are possible.

a) no, b) use of just one antipsychotic, c) use of more than one antipsychotic, d) no use of other class of psychotropic drug, e) use of other class of psychotropic, f) polypharmacy (psychotropics and/or other medication), g) high dose of antipsychotic, h) low dose of antipsychotic, i) high severity of challenging behaviours, j) mild ID, k) moderate ID, severe or profound ID, l) male gender, m) female gender, n) living with family, o) presence of side-effects during the use, p) firts attempt to discontinue the use, q) failure in previous attempt to discontinue, r) other / if yes: specify

**Q22.** At what percentage of all antipsychotic drug users of your patients with ID did you decide not to discontinue the antipsychotic use because there was a registered indication for the prescription.

a) 0%, b) 1-25%, c) 26-50%, d) 50% and over, e) don’t know / no data available, f) other, if yes: specify.

**Q23.** If there was a registered indication was the psychiatric diagnosis for this indication recently (within the foregoing 2 years) confirmed?

a) yes, b) no

**Q24.** By whom was this psychiatric diagnosis made or confirmed?

a) by myself, b) by a colleague within my own professional discipline, c) by a (consultant) psychiatrist, d) the diagnosis was written in the patient care plan, e) the diagnosis was provided by the family, care professional or patient self, f) the diagnosis was written in the patient’s medical file, g) other / if yes: specify

**Q25.** How confident were you about the psychiatric diagnosis of the patients with ID using antipsychotics and where you decided NOT to discontinue the use? (Ratings 0-5; the more confident the higher the rating).

**Q26.** Did you try to discontinue other psychotropics, beside or instead of antipsychotics in your patients with ID?

a) yes, specify, b) no

**Q27.** Did you learn from scientific research, literature, guidelines or education to prescribe less likely antipsychotics for challenging behaviours in people with ID?

a) yes, b) no, c) no difference

**Q28.** Did the publication of the Dutch multidisciplinary guideline on problem behavior in adults with ID (2019) change your likeliness to prescribe antipsychotics for challenging behaviour in people with ID?

a) yes, b) no, c) no difference

**Q29.** Did the entry into force of the Dutch Act on Involuntary care, in which the prescription of psychotropics to people with ID outside guidelines and registered indications is seen as restrictive practice change your likeliness to prescribe antipsychotics for challenging behaviour in people with ID?

a) yes, b) no, c) no difference

**Q30.** Did the publication of the Dutch guideline and the entry into force of the new Act change your likeliness to prescribe another class of psychotropic drugs instead of antipsychotics?

a) no, b) yes, if so: specify

**Q31.** How successful is your working environment in applying the guideline recommendations and policies of professional’s associations regarding the treatment of challenging behaviours and reducing antipsychotic drug use in people with ID?

a) not at all, b) little, c) much, d) completely

**Q32.** Have initiatives been taken in your working environment, such as information meetings, education and training to apply these recommended policies?

a) yes, b) no

**Q33.** Could you indicate how sure you are about being successful in deprescribing antipsyhcotics for challenging behaviours in people with ID?

Ratings 0-5; not at all sure- completely sure

**Q34.** Could you substantiate your rating in the foregoing question

Open text field, multiple answers allowed

**Q35.** Do you use monitoring instruments to measure the effects of antipsychotics’ withdrawal?

a) yes, b) no

**Q36.** If the answer to Q35 is yes: which instrument (s) do you use?

a) Health of the Nation Outcome Scale-Learning Disabilities (HoNOS-LD, b) Clinical Global Impression scale (CGI), c) Aberrant behavior checklist (ABC), d) Brief Problem Inventory (BPI), e) a self-constructed scale, f) other

**Q37.** Did you develop materials or procedures to facilitate the process of antipsychotics deprescribing, e.g., leaflets or deployment of experts by experience?

Open text field, multiple answers allowed

**Q38.** Is professional support available to facilitate the process of deprescribing antipsychotics in people with ID? Multiple answers allowed.

a) no, b) cooperation of different professional sectors, c) cooperation of different care sectors, d) multidisciplinary teams, e) nurse or social worker, f) psychologist, g) behavioural scientist, h), pharmacist, i) other healthcare professionals, e.g., paramedic professionals, j) other /specify

**Q39.** What are, according to your experiences barriers and problems in implementing recommendations and policies regarding the discontinuation of antipsychotics for challenging behaviours? Multiple answers allowed.

a) authority and competence of the prescriber, b) lack of advices/information regarding a structured way to deprescribe antipsychotics for challenging behaviours in people with ID, c) lack of support by a multidisciplinary team, d) resistance of caregivers to discontinue antipsychotics in their clients with ID and challenging behaviour, e) resistance of family to discontinue antipsychotics in their relatives, f) lack of available non-pharmaceutical alternative treatments, g) lack of advice/support of an expert pharmacist, h), resistance of the medical staff, i) other /specify

**Q40.** What resources could be helpful, in your opinion, to facilitate the practice of deprescribing antipsychotics in your working environment? Select the three most important items.

a) better cooperation of stakeholders in the various care sectors, b) the forming of multidisciplinary teams, c) availability of an ID specialist nurse, d) availability of a pharmacist, e) availability of an ambulant mental healthcare nurse, f) availability of other healthcare professionals, e.g., paramedic workers g) financial support

**Q41.** Could you briefly describe (in 2-3 sentences) what are your experiences in your own practice regarding the pursuit to deprescribe antipsychotics for challenging behaviours?

Open text field

**Q42.** Could you briefly describe (in 2-3 sentences) what do you know about clients’ own experiences regarding the deprescribing of their antipsychotics? If so, what ware these?

Open text field

Q43 and Q44 are not substantive
